# A red flag and a red herring—dual pathology of isolated polycystic liver disease with underlying epithelioid angiosarcoma resulting in recurrent hepatic cyst haemorrhage

**DOI:** 10.1093/jscr/rjaf400

**Published:** 2025-07-03

**Authors:** Michael Yulong Wu, Juanita Noeline Chui, Cameron Gofton, Mahsa Ahadi, Kai Brown

**Affiliations:** Department of Gastroenterology and Hepatology, Royal North Shore Hospital, 1 Reserve Road, St Leonards, 2065, Sydney, Australia; Faculty of Medicine and Health, Northern Clinical School, University of Sydney, 1 Reserve Road, St Leonards, 2065, Sydney, Australia; Faculty of Medicine and Health, Northern Clinical School, University of Sydney, 1 Reserve Road, St Leonards, 2065, Sydney, Australia; Department of Upper Gastrointestinal Surgery, Royal North Shore Hospital, 1 Reserve Road, St Leonards, 2065, Sydney, Australia; Department of Gastroenterology and Hepatology, Royal North Shore Hospital, 1 Reserve Road, St Leonards, 2065, Sydney, Australia; Department of Anatomical Pathology, Royal North Shore Hospital, 1 Reserve Road, St Leonards, 2065, Sydney, Australia; Faculty of Medicine and Health, Northern Clinical School, University of Sydney, 1 Reserve Road, St Leonards, 2065, Sydney, Australia; Department of Upper Gastrointestinal Surgery, Royal North Shore Hospital, 1 Reserve Road, St Leonards, 2065, Sydney, Australia

**Keywords:** polycystic liver disease, hepatic haemorrhage, liver cyst, epithelioid angiosarcoma

## Abstract

Polycystic liver disease (PLD) is an uncommon inherited condition that leads to progressive development of hepatic cysts that can become complicated by rupture, infection, and bleeding. We detail the case of a 53-year-old female presenting with haemoperitoneum secondary to hepatic cyst bleeding on a background of isolated polycystic liver disease. Despite extensive investigations to localize a bleeding source, repeated angioembolisation, and partial hepatic resection, the patient had recurrent hepatic haemorrhage. Posthumous histology of the resected liver revealed malignant epithelioid angiosarcoma, a rare vascular tumour underlying the PLD. This is the first reported case with a dual pathology of polycystic liver disease and primary liver epithelioid angiosarcoma. Infiltrative malignancies should be considered in atypical cases of recurrent hepatic haemorrhage.

## Introduction

Isolated polycystic liver disease (PLD) is a rare autosomal dominant with an incidence as low as 1 in 1 000 000 [1, 2]. Whilst often asymptomatic, it can lead to complications, such as cystic rupture, haemorrhage, and infections. Angiosarcomas are a rare, highly aggressive endothelial-cell malignancy of lympho-vascular origin with limited treatment options.

We present the first reported case of concurrent isolated PLD and underlying epithelioid angiosarcoma. Dual liver pathologies can pose significant diagnostic challenges, particularly in acute settings. In recurrent hepatic bleeding without an identifiable source, an underlying malignant infiltrative process should be suspected. When complicated PLD and hepatic malignancy co-exists, curative treatment is often not feasible and early palliation should be prioritised.

## Case presentation

A 53-year-old female with a history of PLD, prior hepatic cyst fenestration, and cerebral palsy presented to a tertiary trauma and high-volume liver surgery centre with acute abdominal pain and haemoperitoneum. She was hypotensive, tachycardic, febrile and had a distended abdomen with diffuse tenderness and guarding. Serology showed severely low haemoglobin level (79 g/L) and leukocytosis (29.6 × 10^9^/L) without coagulopathy.

**Figure 1:**
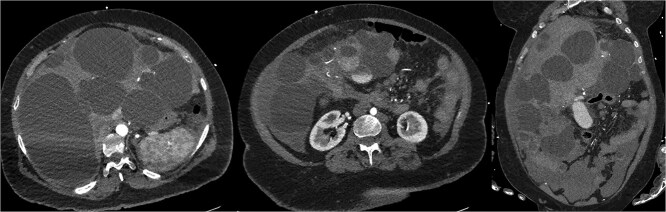
CTMA with axial and coronal views demonstrates a massively dysmorphic polycystic liver with haemoperitoneum and heterogeneously hyperdense material near inferior aspect of liver.

CT mesenteric angiogram (CTMA) revealed no active contrast extravasation but showed large volume haemoperitoneum consistent with a ruptured hepatic cyst, suspected to arise from segment 4b (Fig. 1). The patient was stabilized with broad-spectrum antibiotics, three units of packed red blood cell transfusions and transferred to ICU. Interventional radiology performed prophylactic embolization of segment 4b arteries with concurrent drainage of large-volume blood-stained ascites.

The patient acutely deteriorated on day 7, with abdominal pain recurrence, reduction in haemoglobin (79 g/L), and radiographic evidence of haematoma recollection adjacent to the right liver lobe. With ongoing blood-stained outputs, tachycardia, and continued transfusion requirements, a multi-disciplinary team (MDT) decision was made to proceed with laparotomy with segmental liver resection for definitive source control.

Surgical exploration included excision of a massive segment 7 cyst, multiple adjacent smaller cysts, and prolonged adhesiolysis for exposure. A ruptured cyst in segment 5/6 with active oozing and adjacent clot-filled cysts was identified, prompting limited resection of that area. Intraoperative ultrasound showed no focal mass lesion.

**Figure 2:**
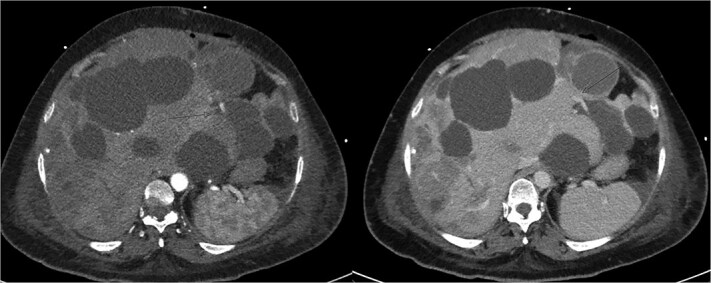
Repeat CTMA with arterial enhancement within the left hepatic lobe (arrow, left image), appearing more ill-defined with contrast pooling on delayed phase (arrow, right image) suggestive of active haemorrhage.

The patient initially improved but deteriorated on post-operative day 4. Repeat CTMA demonstrated active haemorrhage in segment 2—remote from the operative field—and ischaemic changes in segments 6/7, despite a patent portal vein and hepatic artery inflow (Fig. 2). Despite resuscitative measures there were increasing vasopressor requirements, leukocytosis, and deteriorating liver function tests (bilirubin 135 μmol/L, ALP 667 μ/L, GGT 551 μ/L, ALT 234 μ/L, AST 535 μ/L). Differential diagnoses included biliary sepsis, secondary infection of ischaemic liver, ischaemic hepatopathy with progressive liver failure, and angiography complications such as arterial dissection flap. Angiography failed to demonstrate active haemorrhage for an embolization target and CT liver triple-phase showed increased intrabdominal free fluid.

Given the atypical constellation of evolving signs, occult malignancy was considered. Extensive family discussions were held surrounding the patient’s condition without a clear unifying diagnosis. A consensus decision was reached to proceed with re-look laparotomy to assess and treat possible sites of bleeding or infection, acknowledging that this carried a significant perioperative mortality risk. On entry into the abdomen there was an immediate release of 3L of frank blood. The patient became haemodynamically unstable and suffered cardiopulmonary arrest. Cardiopulmonary resuscitation was initiated under supracoeliac aortic cross-clamping, and a massive transfusion protocol was activated. Throughout this period there was ongoing hepatic venous bleeding across multiple sites with attempts at surgical control. Suprahepatic caval control for total hepatic vascular exclusion was attempted but difficult due to a massively enlarged and dysmorphic liver. Median sternotomy for intrapericardial suprahepatic venous clamping and insertion of ventricular pacing wires was performed. Despite over 90 minutes of active resuscitation, it was agreed that with coagulopathy, multifocal and ongoing spontaneous bleeding from the liver, and prolonged cardiac arrest that this was a non-survivable insult and resuscitation was ceased.

**Figure 3:**
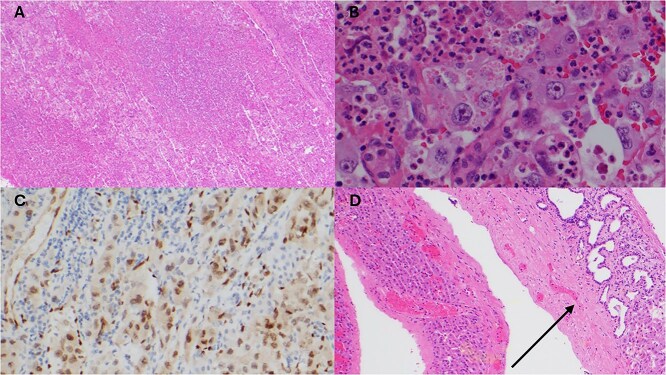
Histopathology of resected liver specimen. (A) Epithelioid angiosarcoma involving liver parenchyma with broad areas of necrosis (4× magnification). (B) Round to polygonal epithelioid tumour cells with prominent nucleoli, and associated red blood cells and neutrophils (40× magnification). (C) ERG immunohistochemistry staining tumour nuclei (20× magnification). (D) Polycystic liver disease, numerous cysts, some with von Meyenburg complexes (arrow) (10× magnification).

Posthumously, the resected liver histology returned with appearances consistent with epithelioid angiosarcoma with deep invasion past the liver capsule (Fig. 3).

## Discussion

This case highlights the diagnostic challenges in identifying aetiology of recurrent, multifocal hepatic haemorrhage. Primary hepatic angiosarcomas are exceedingly rare and often overlooked in differential diagnoses. Their infiltrative nature with lack of a distinct capsule or clear boundary between tissue makes them difficult to detect [3]. In this case, no tumour mass was seen on imaging to suggest an underlying malignancy. Additionally, the presence of PLD with a highly dysmorphic liver complicates the detection of malignancy, both radiologically and on macroscopic inspection at the time of surgery. Immunohistochemistry is crucial for diagnosis. In cases of atypical recurrent hepatic bleeding, early consideration of infiltrative malignancy and timely liver biopsy are essential for diagnosis and prognostication.

Hepatic angiosarcomas carry a poor prognosis with limited treatment options and a lack of established guidelines [4]. Evidence for systemic chemotherapy is weak with management typically based on wide-field radiotherapy and radical surgical resection [4–7]. Given the poor prognosis associated with a highly invasive and non-curable malignancy, earlier diagnosis may have allowed more timely discussions about palliative care.

In this case, emergency surgery was necessary due to an acute abdomen with haemorrhagic shock. Angioembolisation is preferred when a focal site of bleeding is identified as this is carries less morbidity than surgery. Whilst embolization is effective in over 80% of adenomas and hepatocellular carcinomas, there is little experience with angiosarcomas [8]. In the presence of life-threatening haemorrhage for bleeding liver tumours, surgical treatment strategies are akin to liver trauma, including definitive haemostasis, packing or formal liver resection.

## Conclusion

This case underscores the diagnostic and management challenges of concurrent PLD with underlying primary hepatic angiosarcoma. Recurrent multifocal spontaneous haemorrhage was the ‘red flag’ and the PLD was the ‘red herring’. There are no published experiences guiding the management of this complex dual pathology. In those with unexplained recurrent liver bleeding, an infiltrative malignant process should be considered. In those with concurrent morbidities of PLD and a bleeding hepatic malignancy, surgery may offer limited benefits. Given the high risk of deterioration, early MDT discussions are recommended to establish diagnosis and guide goals of care.

## Data Availability

The data that support the findings of this study are available on request from the corresponding author. The data are not publicly available due to privacy or ethical restrictions.
